# A big mesenteric rupture after blunt abdominal trauma: A case report and literature review

**DOI:** 10.1016/j.ijscr.2019.06.041

**Published:** 2019-07-09

**Authors:** Christos K. Stefanou, Stefanos K. Stefanou, Kostas Tepelenis, Stefanos Flindris, Thomas Tsiantis, Spyridon Spyrou

**Affiliations:** a Department of General Surgery, General Hospital of Ioannina “G. Chatzikosta”, Makriyianni Avenue 1, 45001 Ioannina, Greece; b Department of Surgery, Filiates General Hospital, Mpempi 1, 45600 Filiates, Greece

**Keywords:** Blunt abdominal trauma, Small bowel trauma, Mesentery trauma, Urgent laparotomy

## Abstract

**Introduction:**

A blunt abdominal trauma especially in organs less commonly injured (such as small bowel and mesentery injury), are difficult to diagnose.

**Presentation of case:**

We report a case of a blunt abdominal trauma, in a 43 year old male presented in the Emergency Department after a truck vehicle accident. He sustained a chest injury, a pelvic fracture and diffuse abdominal tenderness. The patient had tachycardia (120 pulses/min) and normal blood pressure (120/90 mm Hg). The computed tomography (CT) showed only free fluid. We placed two chest tubes (due to pneumothorax and hemothorax at both sides) and the patient went to the operating room (OP). An external pelvic osteosynthesis was performed first and then we did an exploratory laparotomy, which revealed a big mesenteric rupture. Finally, an enterectomy (circa 2 m) with a fist stage side to side anastomosis was performed.

**Discussion:**

Mesentery and bowel injury constitutes 3–5% of blunt abdominal injuries. The main diagnostic challenge is to identify lesions that require surgery. Diagnostic delay over 8 h can lead to high morbidity and mortality rates. Laparotomy is the standard of care in hemodynamically unstable patients.

**Conclusion:**

In polytrauma cases with abdominal pain and unclear CT findings the decision to proceed with exploratory laparotomy is better than a conservative treatment, because any surgical delay can lead to severe complications.

## Highlights

• In a polytrauma patient with a blunt abdominal trauma and unclear imaging studies the urgent laparotomy is a better choice then wait and see.
• Intestinal and mesenteric injuries are less common and due to diagnostic difficulties they can result in therapeutic delay with severe complications.
• The preservation of the ileo-cecal is an important approach.

## 1 Introduction

Intestinal and mesenteric injuries are less common than solid organ injuries (liver, spleen) and due to the diagnostic difficulties they can result in therapeutic delay.

The intestinal injuries include hematoma, seromuscular tear, perforation and ischemia. Clinical and radiological signs can be obvious for mesenteric and intestinal injuries, but some injuries may present muted or subclinical signs only.

In abdominal traumas hollow organ and mesenteric injuries are found in 3–5 % [[1], [2], [3]] of the patients. They are third in order of frequency after liver and splenic injury [4]. These lesions occur as a result of high energy trauma, like motor vehicle accidents in 70–90% of the cases [[1], [2], [3], [4], [5], [6]]. The work in this case has been reported in line with the SCARE criteria [7].

## 2 Case presentation

**Fig. 1:**
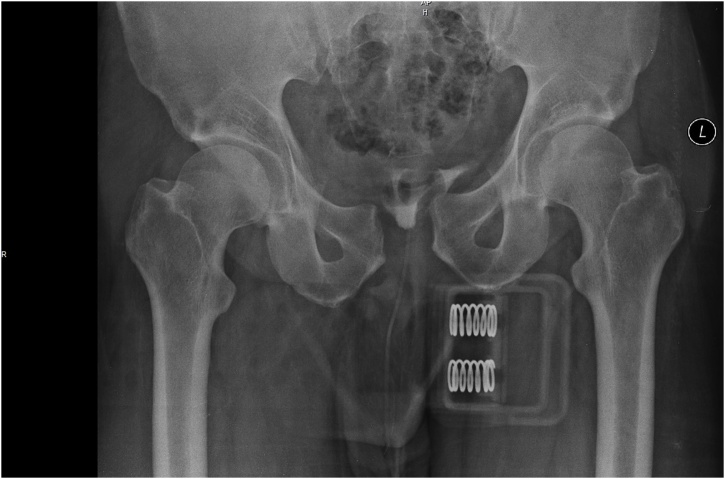
Open book pelvic fracture.

**Fig. 2:**
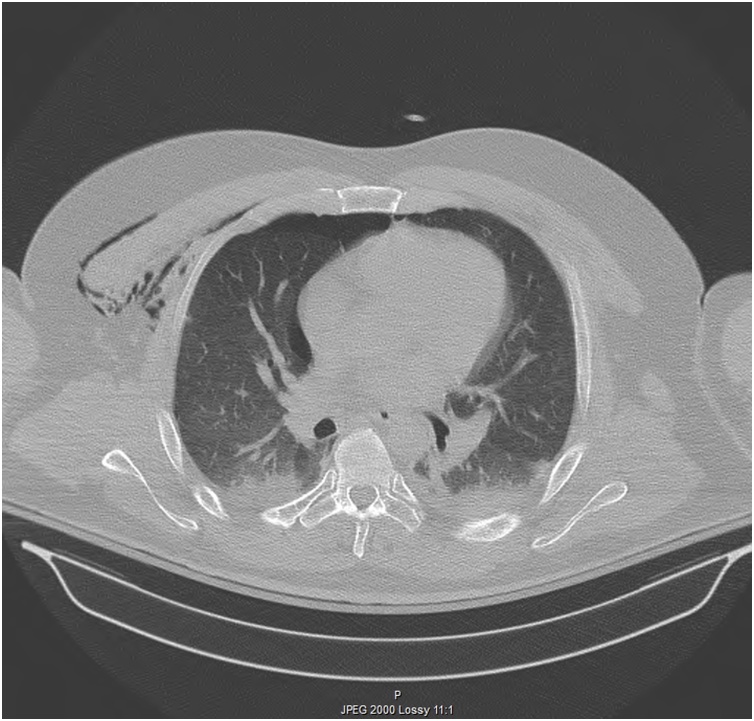
Hemothorax at both sides and right pneumothorax.

**Fig. 3:**
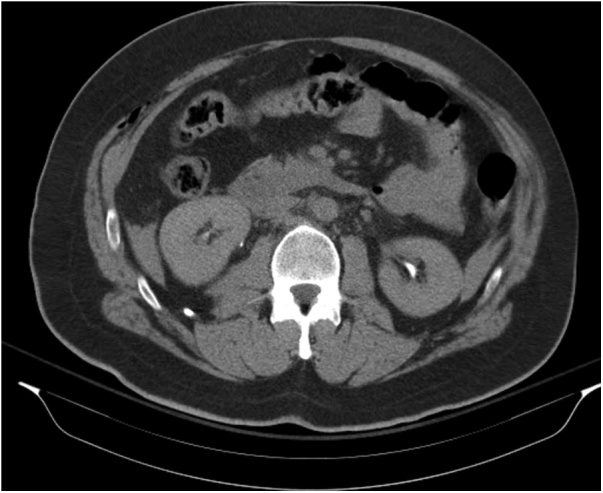
CT abdomen.

**Fig. 4:**
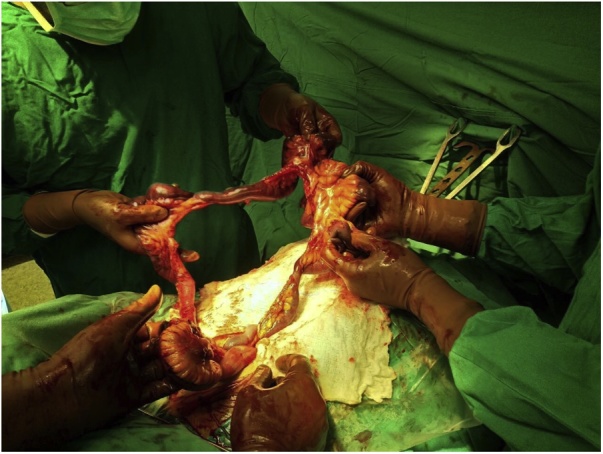
Big mesenteric rupture at the surgery.

A 43-year-old male involved in a roll over truck accident, who wasn’t wearing a seat belt, arrived 4 h after the accident to the Emergency Department of our hospital. He had a blunt abdominal injury, broken ribs in his chest, a right pneumothorax, hemothorax at both sides and an open book pelvis fracture (Fig. 1, Fig. 2). The patient presented hemodynamically unstable with tachycardia 120 pulses/min, BP 120/90 mmHg 23 breaths/min and GCS 15/15. Two chest tubes were placed draining hemothorax at both sides (100cc from the right side and 200cc from the left side), as well as a pelvic binder was placed to stabilize the fracture (Fig. 2, Fig. 3). The abdomen was fatty without wounds or bruises and diffuse tenderness without guarding. A complete blood count was normal with Hb 12,5 mg/dl. An abdominal CT was performed, revealing free fluid without pneumoperitoneum (Fig. 4). Analgesic medication was given, and a serial clinical monitoring was conducted. The patient went to the operating room 6 h after the accident and an external pelvic osteosynthesis was performed. After the osteosynthesis, the patient remained hemodynamically unstable and an emergent laparotomy through a midline incision was performed. An amount of free bloody fluid was found. The small bowel was carefully inspected from the ligament of Treitz up to the ileocecal valve. A big rupture of the mesentery was found and a big segment of the small bowel (circa 2 m) was ischemic (Fig. 1). An enterectomy was performed as well as a side to side anastomosis with a stapler at 6 cm from the ileocecal valve. The peritoneal cavity was washed-out with warm saline solution, and one Jackson-Pratt drain was placed in the peritoneal cavity (in the Douglas pouch). The patient was transferred to the icu. No postoperative complications were recorded. The drain was removed on the 7th day. The patient was discharged home after 30 days. After one-year follow-up the patient is asymptomatic and without complications.

## 3 Discussion

Mesenteric and hollow organ injury represent 16% of all blunt abdominal traumas and occur as a result of high energy trauma involving motor vehicle accidents in 70–90% of cases [4]. Intestinal injuries and their mesenteries (mesocolon and mesentery) are considered as a single clinico-anatomic entity. Small intestinal injuries constitute more than half of all blunt intestinal injuries, with equal involvement of the jejunum and ileum. The second most frequent location of injury is the colon: some studies show that left colon is more commonly injured than the transverse or right colon [5,6]. Duodenal lesions are less common, representing 10% of the total and are often associated with pancreatic trauma. There are several types of intestinal and mesenteric injuries. The mesenteric injuries range from bruising, to hematoma, to frank bleeding through the torn peritoneal envelope. Mesenteric disinsertion may occur with avulsion of the proximal or distal mesenteric root, which may cause intestinal perforation along the mesenteric surface of the bowel and localized devascularization of an intestinal segment resulting in ischemia and secondary perforation. The three main mechanisms leading to these injuries are direct impact, deceleration and increased luminal pressure.

As far as the diagnosis is concerned, history and clinical examination play an important role. History helps to analyze the dynamics of the accident: intestinal or mesenteric injury should be suspected in all high energy blunt trauma. For hemodynamically unstable patients urgent abdominal exploration is required. For hemodynamically stable patients physical examination is important but is often sub-optimal. The presence of abdominal skin bruising means there is a high risk of underlying bowel injury. Seat belt bruising is associated with intra abdominal injury in 20–60% of cases [8,9] and increases the risk of perforated bowel injury by a factor of 2,4 [10]. However, when bruising is an isolated finding with no associated abdominal pain or guarding, the risk of intra-abdominal injury is low [11]. Physical examination should assess abdominal tenderness and guarding, but these signs are non specific since they can be observed with all abdominal injuries. FAST in unstable patients, is not useful for the diagnosis of lesions of intestinal and mesenteric injury because of its low sensitivity [12]. For hemodynamically stable trauma patients CT plays an important role. Radiological signs associated with intestinal and mesenteric injuries are well defined [13]. Signs of intestinal wall injury include: discontinuity of the intestinal wall, thickening of the bowel wall and increased or decreased enhancement of the intestinal wall defect after i.v. contrast injection. Images suggestive of mesenteric injury include: i.v. contrast extravasation (blush) or abrupt discontinuation along a vascular branch, infiltration of mesenteric fat and hematoma. Other signs include: pneumoperitoneum, free peritoneal or retroperitoneal fluid and injury of the muscular layers of the abdominal wall and subcutaneous tissue. With modern multi-slice helical CT scans, the risk of false-negative has become very low, or even zero in two recent studies [14,15]. For those patients with isolated free peritoneal fluid with no evidence of solid organ, intestinal or mesenteric injury, studies reports that 27% of cases are in need of exploratory laparotomy [16].

There are a lot of difficulties to characterize the lesions that require surgical repair and in order to avoid operative delay, surgical exploration is carried out systematically for the least suspicion of intestinal and mesenteric injury. Surgical intervention for all hemodynamically stable patients with suspected bowel or mesenteric injury has led to a high rate of non-therapeutic laparotomy up to 44% for intestinal lesions and up to 31% for mesenteric injuries [17].

The decision to restore intestinal continuity depends on patient condition as well as the degree and duration of intestinal contamination. In most cases, one stage repair or resection with restoration of continuity can be performed for both small intestinal and colonic lesions [18]. If there is any delay in surgical intervention, a stoma is preferable, especially in cases of colonic perforation. Proximal jejunal lesions pose a specific problem, because ostomies at that leve result in high flow and lack of nutritional absorption. It is also important to preserve, if possible the ileocecal valve, because there are many benefits including prevention of small bowel overgrowth, diarrhea (electrolytes imbalance) as well as preserving colonic absorptive surface capacity. In complex situations, or in elderly patients with co-morbidities, a two stage technique with initial intestinal exclusion at the first DCL and restorative anastomosis at the “second look” may be a solution.

The management of blunt abdominal trauma has evolved over time. While laparotomy is the standard of care in hemodynamically unstable patients, stable patients are usually treated conservatively. Laparotomy is associated with high morbidity as well as additional surgical trauma if it is unnecessary. Studies disclose a high morbidity up to 413% in such cases [19]. The laparoscopic approach allows us to identify a bleeding or the presence of visceral lesions combining the minimally invasive treatment of the possible injury and reduces the incidence of negative laparotomies [20]. In hemodynamically stable patients who don’t suffer from intracranial injuries, high grade chest trauma, preexisting intraabdominal adhesions as well as pregnancy, the laparoscopic approach can be diagnostic and therapeutic [21,22].

Laparoscopic repair of the diaphragm is the most common therapeutic minimally invasive procedure in blunt and penetrating trauma [23]. Diaphragmatic injuries can often be undiagnosed, due to the physiological movements of the diaphragm, and can be revealed during surgical procedures. Only approximately 5% of patients with abdominal trauma have a diaphragmatic injury mainly caused by blunt abdominal trauma of the chest and abdomen (75%), and more rarely by stabbing (25%) [24,25].

The advantages of laparoscopy in trauma are: the reduction of blood loss, the low risk of postoperative pain, the low risk of contamination, the early functional recovery and the better cosmetic result.

The mortality in the present series of intestinal and mesenteric trauma is between 10–23%. Patient prognosis is affected by the delay interval between the time of injury and the time of therapeutic intervention. In many studies the threshold varies from 5 to 8 h. [[26], [27], [28], [29]].

## 4 Conclusion

Mesenteric and bowel injuries are difficult to diagnose and their undiagnosed complications are severe with high mortality rates. In a polytrauma patient, with abdominal symptoms, clinical examination and imaging studies (preferable a ct scan) are necessary. There are radiological signs that are associated with the mesenteric and bowel injuries but sometimes these signs are unclear in polytrauma patient. If you have a polytrauma patient with abdominal tenderness, it is better to think for exploratory laparotomy as soon as possible rather to wait and see. The preservation of the ileocecal valve is also an important approach, because of the benefits, including preservation of colonic absorptive surface capacity and electrolyte balance.

## Conflicts of interest

There are no conflicts of interest.

## Sources of funding

The study received no financial support.

## Ethical approval

The patient has provided permission to publish these features of this case.

Ethical approval not required.

## Consent

Written consent from the patient has been obtained.

## Author’s contribution

All the authors have read the manuscript and have approved the submission.

Author contribution:

- C. Stefanou, concept and design of study, manuscript preparation.
- S. Stefanou, acquisitition and interpretation of data, literature search.
- K.Tepelenis, revising the article critically for important intellectual content, manuscript editing, assisted with the operation.
- S. Flindris, revising the article critically for important intellectual content, manuscript editing, assisted with the operation.
- T. Tsiantis, revising the article critically for important intellectual content, manuscript editing, assisted with the operation.
- S. Spyrou, final approval of the version to be published, performed the operation.

## Registration of research studies

N/A.

## Guarantor

I Christos Stefanou am responsibly for this case.

## Provenance and peer review

Not commissioned, externally peer-reviewed.
